# Fracture of the Ulna during Massage Following Operation for Colles’ Fracture*Read before the Clinical Society of the New York Polyclinic Medical School and Hospital stated meeting, held February 5, 1906. The President, Dr. J. J. MacPhee, in the chair.

**Published:** 1907-02

**Authors:** 


For Texas Medical Journal.

### Fracture of the Ulna During Massage Following Operation for Colles' Fracture.*

Dr. F. C. Keller showed this patient. After the removal of the splint her arm was being massaged when the ulna snapped at a point several inches above the original site of fracture. This occurred six or eight weeks after the operation, and could be attributed only to some inherent disease of the bone.

Dr. J. C. Robertson said that he had put up from 200 to 300 cases of Colles' fractures during the past eight years, and until two years ago had applied a posterior splint, the arm being semiflexed in a stiff position. This gave far from perfect results in eight out of ten cases. A careful study of these cases had convinced him that the best results were obtained by applying a posterior splint from the hand to the elbow, keeping the arm perfectly straight, and putting a pad of cotton under the wrist. As a result there is no sharp protrusion of the ulna at the elbow, as often occurs when the hand is put up anteflexed.

Dr. J. A. Bodine said that something was wrong with the composition of the bones of this patient, as ulnas do not snap from massage. The bones were probably chalky. As to Dr. Robertson's experience with Colles' fractures, he thought that each surgeon favored the line of treatment with which he, personally, had ob-

---

*Read before the Clinical Society of the New York Polyclinic Medical School and Hospital stated meeting, held February 5, 1906. The President, Dr. J. J. MacPhee, in the chair.

tained the best results. He thought a posterior splint more practical, for the reason that the posterior surface of the arm is a straight surface, and has no cutaneous nerves and no return blood supply. If a rigid anterior splint is applied to the front of the arm, edema is caused by obstruction of the circulation.

### INTUSSUSCEPTION.

Dr. Alexander Lyle reported a case of intussusception occurring in a child seven and one-half months old. The patient, well-nourished, healthy, active and breast-fed, had enjoyed perfect health, with the exception of constipation, until the evening of December 18, 1905, when he was suddenly seized with severe abdominal pain, as evidenced by crying and flexion of the thighs upon the abdomen. He was given a hot mustard footbath, and, internally, hot water with gin and paregoric. His bowels had moved normally on the preceding day, but not on the day of the attack. At 1 a. m. the child passed about half an ounce of bloody mucus, but no fecal matter. Pain was severe and recurrent in character, and at 6 a. m. on the 19th a physician was summoned. He ordered half an ounce of castor oil. This failed to produce an evacuation of the bowels. On the evening of the 19th he ordered an enema (rectal) of glycerine and hot water. During the night the mother noticed a sudden change in the child's condition and thought it to be dying. She could not reach the physician, and in the morning the speaker was summoned. On reaching the house he found that the physician had arrived and had given an enema of an ounce of castor oil and one pint of warm water, the water returning with bloody mucus.

Hasty examination showed a state of collapse, a weak pulse that could not be counted, a tense, rigid abdomen, and a rectal temperature of 103° F. Diagnosis of intussusception was made, and immediate operation advised as offering the only hope (and that a poor one).

The child was immediately brought to the Polyclinic Hospital and operated upon. No tumor could be mapped out, even after he had been anesthetized. An incision was made in the right rectus muscle, just below the umbilicus, the abdominal contents examined and intussusception located in the ileocecal region. A firm, dense band of adhesion anchored this portion of the intestine, necessitating a considerable amount of work before it could be brought into the wound. This was finally accomplished and the intussusception reduced. The gut was not gangrenous and therefore was returned to the abdominal cavity. A loop of small intestine was picked up and two drams of saturated solution of magnesium sulphate was thrown into it by means of a syringe, the needle of which was carried obliquely into the lumen, the object being to evacuate the bowels as soon as possible. The abdominal wound was then closed.

Following the operation the child's temperature rose to 103.5° F., and remained so until 1 a. m. of the next day, when it dropped gradually to 99.5° F., and did not rise above 100.8° F. at any time afterward. The pulse could not be counted until the temperature had fallen to 101.8° F., when it was 160, later falling to 118 or 120. The bowels moved five times during the first twenty-four hours after the operation.

The speaker emphasized the point that valuable time must not be lost by useless, or, more properly speaking, positively injurious and dangerous medication. The sudden abdominal pain, followed by a discharge of bloody mucus from the rectum, the recurrent attacks of pain and absence of fecal evacuations indicate immediate operation. Gangrene or extensive adhesions, or both, are produced by delay, and an intestinal resection and circular enterarrhaphy will be necessary. An early operation, on the contrary, enables the surgeon to early effect reduction.

Dr. Bodine said that one point should be emphasized in the diagnosis of an inflammatory abdominal condition in a child, and that is the expression of the face, which is always typical. Another aid is the abdominal pain. He thought it would have been impossible to have made a differential diagnosis between this condition and appendicitis if it had not been for the presence of the bloody mucus.

Dr. Maurice Packard said that in cases of abdominal lesions in children up to 3 years of age, the differential diagnosis between intussusception and strangulated hernia usually has to be made. The only point in diagnosis especially pointing to intussusception is the bloody mucus. A body temperature of 103° F. and a rapid pulse are also significant, as the statement is made in many text-books that, except in appendicitis and general peritonitis, the temperature and pulse are normal and the abdomen relaxed. It has been his experience that in intussusception children always have a high temperature and have a pulse so rapid that it is almost impossible to count it. In cases of intestinal obstruction the absence of stools and gas assists one in making a differential diagnosis, as in intussusception only mucus and blood pass from the bowels.

### LARGE OVARIAN CYST.

This specimen was presented by Dr. C. G. Child, Jr. He had removed it from a patient 38 years of age. She had complained of pain during the previous four or five years, during which time she noticed the presence of a tumor, which grew progressively larger. Examination revealed an enlargement reaching to the umbilicus. It was impossible to palpate the appendages on either side, and it was also impossible to determine on which side the tumor originated. On account of the pain being on the right side it was concluded that the tumor was of the right ovary, but at the time of operation it was found to involve the ovary on the left side and to have rotated the uterus. It firmly compressed the appendages on the right side, which accounted for the pain on that side. A transverse incision showed the cyst to be inherent in all directions to the omentum and posterior peritoneum. A portion of it was free from adhesions, and at this point the fluid contents were aspirated. The sac was then pulled out, with the intestines and omentum, and the adhesions separated. The sac contained a dark, water-like fluid, which is rather unusual, the contents of such a tumor usually being of a yellow straw color.

The paper of the evening was by Dr. J. C. Taylor, and was entitled

### ACUTE PELVIC INFECTIONS.

He said, in part: It is but a few years since a woman's tubes and ovaries were sacrificed by an operator lest a future laparotomy should be required. Actuated by a sense of thoroughness, he deprived women of the function of menstruation, which is interwoven with their mental as well as physical life. It is better to conserve these organs, even if elaborate and hazardous procedures must be adopted to accomplish this end as well as to cure the patient. He did not advocate, however, the carrying of conservatism in connection with special organs so far as to endanger the constitutional condition of women. There is a broader conservatism, which seeks to restore the general health of the patient, even if special organs must be sacrificed to attain such an end. To this end he made an appeal for early surgical interference in the acute diseases of the female organs. Conservative operations sometimes may fail; but even if they do, radical procedures must be adopted later without added risk to the patient. On the other hand, it is impossible to restore organs removed by radical work.

For many years it has been customary in most large hospitals to treat patients suffering from extension of gonorrheal inflammation to the tubes by hot antiseptic douches or perhaps by tampons and an ice-bag externally over the lower abdominal region. When the acuteness of the attack had somewhat subsided the tubes as well as the ovaries were frequently swollen and engorged to such an extent as to be designated as tumors, and removal was advised; whereas, without apparent mutilation, the inflammation might have been checked in the beginning and the woman allowed to keep her organs, though somewhat damaged. The conservative work to be attempted is mainly that of evacuating the free pus in the culdesac when the operator is convinced by the bulging of the wall of the posterior fornix that purulent exudate is present in abundance. The gonococci, in an active state, after they have gained entrance into the uterine cavity, cause a destruction of the superficial cells, work their way into the deeper layers, and are the cause of an immense amount of purulent exudate, destruction and infiltration of the outer layers and edema of the deeper structures. Unfortunately, after gonorrhea has once become well established within the uterus, it invades by continuity of tissue the Fallopian tubes. The inner surface of the uterus may become such an active seat of inflammation in its deeper layers that the walls of the smaller vessels become involved, as do the surrounding lymphatics, and the normal structure is almost entirely destroyed. The walls of the uterine cavity thus become suppurating surfaces, which later become sclerotic, and this is followed by a shrinking of the organs. This is frequently the case in mixed infections.

If the tubes are opened and drained during the onset of the disease, the woman may regain her organs, though somewhat damaged. The operation is very simple, but it necessitates a thorough knowledge of female pelvic anatomy and careful manipulation of special instruments. An incision is made on the posterior surface of the cervix at the juncture of the vaginal mucous membrane with the cervical, care being taken to keep close to the cervix. A pair of blunt-pointed scissors, curved on the flat, seems best adapted for this purpose. When the incision is made in the curve of the fornix, a painful scar is apt to result; the nearer the rectum is approached the greater being the sensory nerve supply. After incising the mucous membrane and retracting the divided edges, a small amount of loose alveolar tissue is encountered (most marked in women after the menopause). After incising this the peritoneum is easily divided or punctured. With the forefingers the opening can be enlarged. The utero-sacral ligaments being pushed outward by the palmar surfaces of the fingers and the intestines carried out of the way by means of the Trendelenberg position and held there by pads, the tubes are easily brought into view by means of the proper instruments for retraction. If this procedure is adopted in the very early stages, as it should be, the tube will be found reddened, swollen, and a tendency to sink into the culdesac. It should be grasped with a pair of blunt forceps, such as those of the modified Hunter type, on the dorsal surface, and pulled into the opening. It should be remembered that the tube, like the ovary, except at its uterine extremity, is fed by small ascending branches from the ovarian artery, which enter the structure from the lower surface; consequently, when an incision is made it should be on the opposite side. Care should be taken to keep the intestines out of the way by means of pads, the tubes being incised along the outer two-thirds of the upper border. The contents should then be evacuated and the entire surface thoroughly swabbed with 5 per cent iodoform gauze. At first there will be considerable oozing of blood, which gradually subsides, no main vessel having been cut. A small strip of iodoform gauze should then be placed over the raw surface, an end protruding into the vagina. The first effect of this treatment is to reduce the intestinal cellular infiltration, as it is a well-known fact that gonococcus does not thrive well on exposed surfaces, its natural abode being in the deep recesses of compound racemose glands. The gauze may be removed from the culdesac in from five to six days. This may be done with safety after such a period, as the life of the gonococcus at best is very short, except in racemose glands and closed sacs.

